# Spontaneous Intracranial Hypotension: Case Report and Update on Diagnosis and Treatment

**DOI:** 10.3390/diagnostics14090881

**Published:** 2024-04-24

**Authors:** Maria Carolina Jurcau, Anamaria Jurcau, Vlad Octavian Hogea, Razvan Gabriel Diaconu

**Affiliations:** 1 Faculty of Medicine and Pharmacy, University of Oradea, 410087 Oradea, Romania; jurcau.mariacarolina@student.uoradea.ro (M.C.J.); hogea.vladoctavian@student.uoradea.ro (V.O.H.); diaconu.razvangabriel@student.uoradea.ro (R.G.D.); 2 Department of Psycho-Neurosciences and Rehabilitation, University of Oradea, 410087 Oradea, Romania

**Keywords:** spontaneous intracranial hypotension, cerebrospinal fluid leak, diagnostic criteria, magnetic resonance imaging, myelography, epidural blood patch

## Abstract

Spontaneous intracranial hypotension (SIH) is an important cause of daily headaches that occur in young and middle-aged, active persons and is often misdiagnosed, leading to prolonged inactivity and rather high healthcare expenditures. Its diagnosis requires a high degree of clinical suspicion and careful interpretation of imaging studies. We present a case of SIH, which was successfully treated but which posed serious diagnostic challenges, ranging from cerebro-vascular disease and meningitis to granulomatous diseases, and for whom every therapeutic attempt just worsened the patient’s condition until we finally reached the correct diagnosis. To raise awareness of this condition, we also present an updated overview of the clinical picture, evaluation, and treatment options for SIH.

## 1. Introduction

Spontaneous intracranial hypotension (SIH) is an important cause of daily headaches, mainly among young and middle-aged adults [1]. The annual incidence is higher than previously thought, varying between 3.7 and 5/100,000 persons [2], which is about half of the incidence of aneurysmal subarachnoid hemorrhage [3]. Nonetheless, it is still considered a rare condition, although, given the more widespread use of imaging techniques in patients with headaches, the incidence of diagnosed SIH is increasing [4]. Females are affected about twice as often as males [5], the peak incidence being between 30 and 50 years [6].

Headache is one of the most common complaints in patients presenting to the primary care physician, ED, or referred to a neurologist [7]. Although in some situations, the diagnosis and etiology of headache are straightforward, the large majority of patients with intracranial hypotension initially receive an incorrect diagnosis, and the delay to the correct diagnosis can range from several days to weeks and even years [8]. For this reason—we present a case of spontaneous intracranial hypotension that posed serious diagnostic challenges and further discuss the clinical presentation and management of this condition, with the aim of raising awareness of this condition and avoiding diagnostic delays in patients presenting with a similar clinical picture.

## 2. Case Report

This study was in accordance with the 1964 Declaration of Helsinki or the institution’s ethical standards, subject to informed consent obtained from the patient.

### 2.1. Clinical Data

A 51-year-old male patient presented to the ED for a headache over the past 7 days followed by double vision. He worked as a carpenter. Prior medical history: hypertension, asthma, and chronic maxillary sinusitis with a flare-up in the past week manifested with bloody nasal discharge. His chronic medication consisted of 5 mg Perindopril, 1.25 mg Indapamide, and intranasal Levocetirizine. Given his prior history of hypertension and the sudden onset of diplopia, he was admitted with a suspected vertebro-basilar stroke.

Upon general physical examination, his blood pressure was 146/86 mm Hg, rhythmical heartbeats were 82/minute, and temperature was 36.6 degrees Celsius; he was slightly overweight (BMI 26.8 kg/m^2^), with no audible bruits on auscultation of the carotid and vertebral arteries.

The neurological examination revealed slight neck stiffness, convergent strabismus with a limited abduction of the left eye, preserved muscle force in all four limbs, normal tendon reflexes but bilaterally indifferent cutaneous plantar responses, and normal general somatic sensation, coordination, and speech.

Prior to his admittance to the neurology ward, he had an ophthalmological examination, which found preserved vision and normal aspect of the optic fundus in both eyes and assessed the intraoptic pressure—14 mm Hg in the right eye and 11 mm Hg in the left one.

### 2.2. Initial Evaluation and Treatment

Biochemical and hematological analyses showed normal CBC and ESR, normal liver and kidney function, glycemic levels of 104 mg/dL, and a C reactive protein of 3.70 mg/L (normal range 0–5.0).

**Figure 1:**
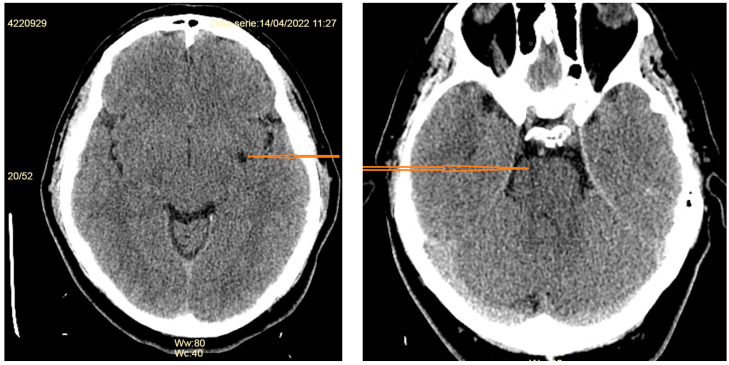
Non-contrast-enhanced CT scan of the patient on admission, showing a lacunar stroke in the left capsulo-lenticular area and the right pons (red arrows).

A non-contrast-enhanced computed tomography (CT) scan described a left capsulo-lenticular lacunar stroke and a similar lesion in the right pons (Figure 1).

After standard medical treatment for the suspected stroke with Aspirin 100 mg/day, Atorvastatin 80 mg/day, and blood-lowering agents, the next day, the left-sided abducens palsy was complete, and abduction was limited in the right eye as well. A lumbar puncture was performed, and the CSF was analyzed, revealing a protein content of 78.6 mg/dL (normal range 20–40 mg/dL), glucose content of 67 mg/dL, and chloride content of 121 mmol/L, as well as 9 mononuclear cells. It is not customary in our ward to measure the opening pressure of the CSF, but it appeared normal. Following the lumbar puncture, the patient complained of worsening headache with nausea. This was ascribed to the lumbar puncture, and bed rest was advised, but we also feared a cerebral venous thrombosis, which is why anticoagulants were added and an MRI was scheduled.

**Figure 2:**
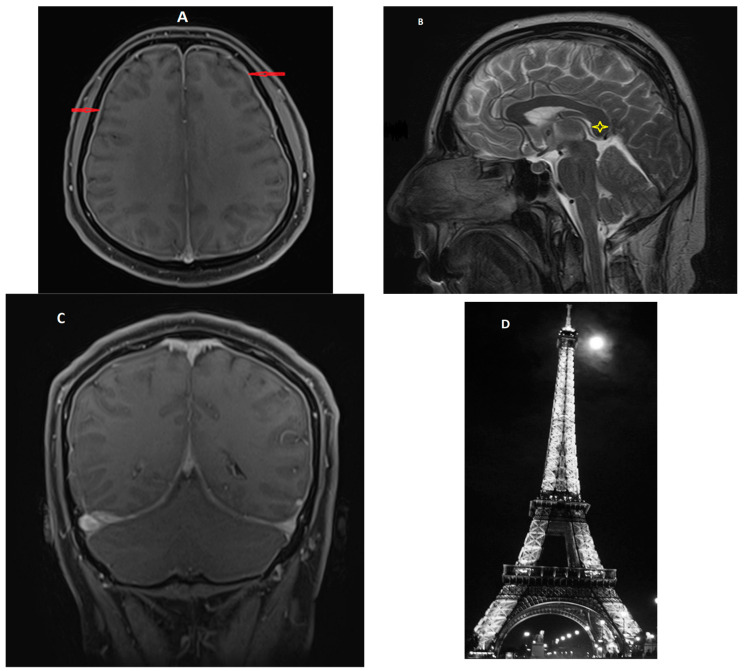
Initial brain MRI of the patient. (**A**) Axial contrast-enhanced T1-weighted image showing diffuse pachymeningeal enhancement (red arrows). (**B**) Sagittal T2-weighted image showing signs of brain sagging (slight downward displacement of the splenium of the corpus callosum, droopy penis sign—yellow star). (**C**) Coronal contrast-enhanced T1-weighted image showing enhancement of the pachymeninges (“Eiffel by night” sign) and engorgement of the sigmoid sinus. (**D**) Image of the Eiffel Tower at night.

The contrast-enhanced brain MRI (axial T2, FLAIR, DWI, sagittal T1, coronal FLAIR, and 3D TOF) revealed a normal cerebral parenchyma, with no diffusion restriction, but a diffuse thickening of the meninges with significant enhancement, while the arteries of the circle of Willis had normal position, caliber, and blood flow (Figure 2). In addition, a minimal collection of fluid in the inferior third of the right maxillary sinus was detected. The suggested diagnostic possibility was infectious meningitis. The signs of brain sagging (downward displacement of the splenium of the corpus callosum) were overlooked.

Although the nine mononuclear cells in the CSF did not qualify for a diagnosis of meningitis, after consultation with an Infectious Diseases’ specialist, treatment with IV Acyclovir, IV Ceftriaxone 2 g/day, Dexamethasone 24 mg/day, and Mannitol was started and continued for 2 weeks, with the improvement of the abducens nerve palsies and the headache, following which the patient was discharged.

### 2.3. Follow-Up

Three weeks after discharge, the patient returns with a worsened headache (mainly left-sided hemicrania) and aggravated diplopia. Neurologically, he had complete left-sided and partial right-sided abducens nerve palsy with no motor deficit, normal tendon reflexes, and bilaterally indifferent cutaneous plantar responses.

**Figure 3:**
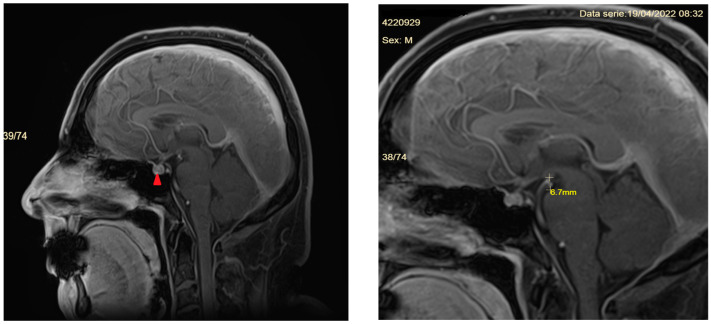
Sagittal T1-weighted contrast-enhanced images of the patient’s brain. The picture on the left shows the pituitary enlargement and hyperemia (red arrow), while the one on the right highlights the reduced mammilopontine distance (6.7 mm). Although normally, this distance is measured in sagittal non-enhanced T1 images we could retrieve only enhanced T1 sagittal sections in the imaging study.

He is screened with a contrast-enhanced CT scan of the chest, abdomen, and pelvis (searching for malignancies leading to leptomeningeal metastases), a second lumbar puncture is performed to exclude tuberculous meningitis (negative), and serum angiotensin convertase is measured to exclude sarcoidosis (18 IU/L, normal range 20–70 IU/L). This time, the CSF protein content increased to 92.2 mg/dL. A second brain MRI is performed, showing a significantly enhancing and thickened supra- and infratentorial meninges extending into the cervical area (Figure 3). Retrospectively analyzing the imaging studies, the patient also showed additional signs, but not as striking as the pachymeningeal enhancement. The mammilopontine distance is the shortest distance between the inferior margin of the mammillary bodies and the superior surface of the pons, measured in mid-sagittal T1-weighted images. It is normally around 7 mm [9] or even 9 mm [10], whereas in our patient it was 6.7 mm. In addition, the pituitary gland was enlarged and hyperemic.

In our view, the main diagnostic possibilities at this stage were idiopathic pachymeningitis or secondary pachymeningitis (either in Wegener granulomatosis—considering the persisting nasal discharge or an IgG4-related one). As such, the patient was treated with pulse therapy with IV methylprednisolone 1 g/day for 3 days, followed by a taper of oral Prednisolone, together with diuretics to prevent increases in blood pressure. Despite no significant improvement, he was discharged after one week with the recommendation to measure the levels of the antineutrophil cytoplasmic antibodies (cANCA and pANCA) as well as the IgG4 levels.

**Figure 4:**
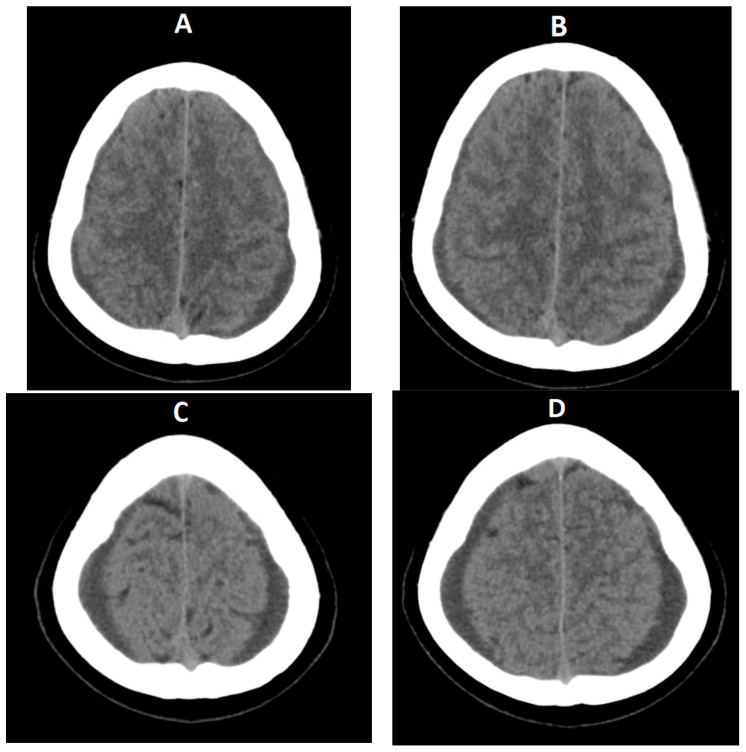
CT scan on third admission (**A**,**B**) and one week later of the patient (**C**,**D**) showing enlarging bilateral temporo-parietal subdural hygromas measuring 5 (**A**,**B**) and 10 mm (**C**,**D**).

The patient returned three days after discharge through the ED complaining of severe headache, tinnitus, nausea, vomiting, and balance disturbances. The CBC revealed hemoconcentration (16,000 leukocytes/mmc, 6.16 × 10^6^ erythrocytes/mmc, 253,000 platelets/mmc, increased blood urea (30 mg/dL, normal range 8.9–20.6 mg/dL), elevated liver enzymes and hyponatremia (122 mmol/L). The CT scan showed a posterior bilateral hygroma with a maximum width of 5 mm and a hyperdense aspect of the falx cerebri. In evolution, after 7 days the hygromas almost doubled in dimensions (Figure 4).

### 2.4. Management

**Figure 5:**
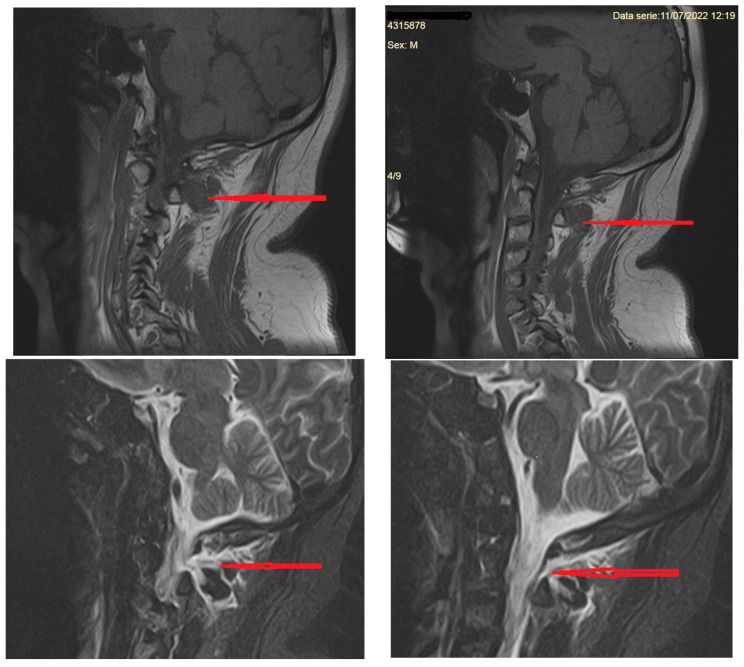
MRI of the cervical spine (T1 FSE sequences in the **upper** row and T2 STIR sequences in the **lower** row)) showing the fluid collection infiltrating paravertebral structures at the C1–C2 vertebral level (red arrows).

It was only after the development of these hygromas that we became aware of the possibility of a CSF leak and SIH as a diagnosis, asking for the help of the Neurosurgery department, which took over the care of the patient. The cervical and dorsal MRI revealed a fluid collection posterior to the C1–C2 vertebrae, judged to be the location of the dural fistula. A lumbar autologous blood epidural patch is performed on 29 June 2022, and bed rest is imposed on the patient. A follow-up MRI of the cervical spine shows an increase in the fluid collection in the soft tissues lying posteriorly to the C1–C2 vertebrae (Figure 5), which is why on 11 July 2022, a cervical epidural blood patch was placed under CT guidance, followed by significant improvement of the patient’s symptoms. Upon detailed questioning, the patient remembers having had a suboccipital puncture performed by a neurosurgeon for a headache 7 years prior to his admittance.

**Figure 6:**
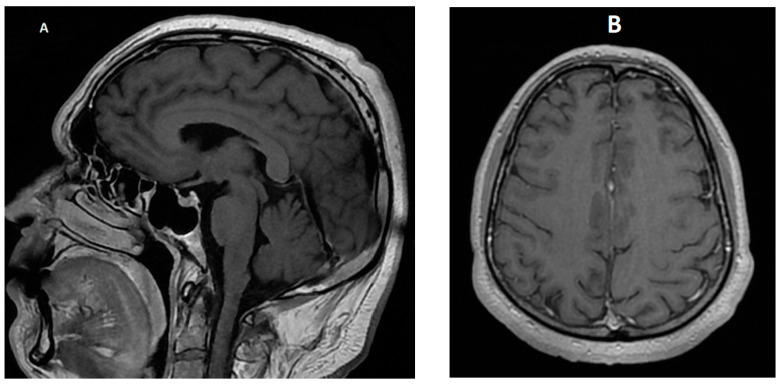
Normal MRI imaging of the brain on follow-up. (**A**)—sagittal contrast-enhanced T1-weighted image; (**B**)—axial contrast-enhanced T1-weighted image.

Neurosurgical and imagistic follow-up after 3 months revealed the complete remission of the hygroma, and the patient was symptom-free (Figure 6).

The patient was referred one year after the CT-guided epidural blood patch to the Neurology clinic for a regular check-up and continued to be symptom-free, having resumed his work. To date (about 22 months from his initial admittance), he has not accessed the Neurology or Neurosurgical departments of our hospital, nor has he presented to the ED.

### 2.5. Discussion

The diagnosis of spontaneous intracranial hypotension requires a high degree of clinical suspicion.

First, the key feature suggesting the diagnosis is orthostatic headache, although, in time, this strict postural relationship tends to fade. Our patient did complain of headache worsening in the upright position, but only after admission and after the performance of a lumbar puncture, when it was labeled as post-puncture headache. A more detailed history may have revealed the presence of orthostatic headache even before the lumbar puncture, as well as the presence of the previous suboccipital puncture. In addition, the abrupt onset of double vision in a patient with vascular risk factors and the presence of lacunes on the initial CT scan suggested a vascular origin of the symptoms, while the prominent abducens nerve palsy (a false localizing sign appearing mostly in intracranial hypertension) and diplopia, as well as the nasal discharge, raised the possibility of sarcoidosis or Wegener granulomatosis, suspicions that delayed the correct diagnosis and treatment.

Second, a more thorough examination of the brain imaging studies could have identified, aside from the pachymeningeal enhancement, the accompanying signs of brain sagging, leading to an earlier diagnosis and prevention of the subsequent complications (hygromas and hyponatremia). Smooth and diffuse dural enhancement is highly suggestive of SIH and should not be confused with meningitis, which involves rather the leptomeninges, while granulomatous diseases, such as polyangiitis or sarcoidosis, show mainly nodular dural thickenings, as do metastases.

On the other hand, such a cranial location of the CSF leak at the C1–C2 vertebral level is quite rare, but in view of the patient’s prior suboccipital puncture, it appears to be related to it. It is unclear why the dural tear became symptomatic so late, 7 years after the procedure. One possibility would be that, given the current flare-up of the sinusitis, the frequent Valsalva maneuvers may have contributed to the reopening of the dura. Alternatively, a minor and rather “compensated” CSF leak in which the postural character of the headache was no longer prominent might have been worsened by our lumbar puncture performed the next day after admission, when we witnessed the patient’s worsening neurological status. However, this possibility tends to be contradicted by the ineffectiveness of the lumbar epidural blood patch. Finally, the diuretics prescribed together with the steroid treatment to avoid increases in blood pressure, may have supplementally lowered the intracranial pressure hastening the disease course and leading to the occurrence of hygromas.

The hyponatremia presented by the patient on his third admission may have been precipitated by the diuretics prescribed but is more likely to be due to a pituitary dysfunction leading to the syndrome of inappropriate antidiuretic hormone secretion (SIADH) in the context of SIH.

We have learned from this case to always assess the images ourselves as well (not just rely on the radiology report) and to actively discuss the evaluation with the radiologists. It is true that a diagnostic possibility is required on the referral letter, but depending on the actual findings, other etiologies for the patient’s symptoms may be evoked by the imaging study, leading to a continuous reassessment of our clinical judgment. Nonetheless, after an almost 3-month delay, the correct diagnosis was finally reached, and the patient was successfully treated, resuming his occupation and normal lifestyle. 

## 3. Update on the Etiology, Diagnosis, and Management of Spontaneous Intracranial Hypotension

Intracranial hypotension can occur either spontaneously or following a lumbar puncture (iatrogenic). The presumed pathogenesis is related to a decrease in the volume of the spinal cerebrospinal fluid (CSF), leading to reduced buoyancy of the brain in the upright position, traction of the dural structures of the posterior fossa, and increased intracerebral blood volume with cerebral venous dilation [11].

Spontaneous intracranial hypotension is most often tied to a CSF leak caused by a disruption of the spinal meninges during a stretch or twist. Alternatively, a tear in the dural nerve sheath [12] may make the protrusion of the arachnoid layer more prone to rupture [13]. Connective tissue disorders may predispose to the formation of meningeal diverticula, which are the leading cause of CSF leaks [14] that occur mainly at the level of the thoracic or lower cervical spine [15]. Factors contributing mainly to ventral dural tears are calcified disc herniations or discogenic microspurs and osteophytes [16]. Less often, other causes for spontaneous intracranial hypotension can be identified, such as CSF-venous fistulas, allowing for spillage of CSF into the circulatory system [17], or bariatric surgery [18].

### 3.1. Clinical Presentation

#### 3.1.1. Headache

The most common symptom is headache, reported by 97% of patients [19]. The headache occurs or worsens upon assuming an upright position, reaches peak intensity usually within 15 min [20], and improves or disappears in the recumbent position [8]. However, in time, this posture-dependent character may become less prominent. Patients describe the headache as occipital, frontal, or diffuse [19], while the severity may vary from mild to incapacitating. Nonetheless, almost 8% of patients do not exhibit this orthostatic characteristic, which is why the ICHD-3 criteria no longer use this criterion [21].

Headache may be accompanied by photophobia and neck stiffness, mimicking subarachnoid hemorrhage [22], leading doctors to perform CT angiographies in search of a ruptured aneurysm.

The occurrence of the headache is thought to be caused by a low volume of CSF that leads to sagging of the brain and traction of the tension-sensitive posterior fossa dura mater and cranial nerves [8,23].

#### 3.1.2. Neck and Back Pain

The incidence of neck pain and neck stiffness varies between 33% [19] and 71% [24]. They occur either concomitantly with the orthostatic headache or precede it by days to weeks [20]. A small proportion of patients (3.2%) may report low back pain [19].

#### 3.1.3. Auditory Disturbances

About 50% of patients with SIH may complain of a wide range of auditory disturbances [23], such as tinnitus (19%), dizziness (14%), vertigo (3.8%), hearing loss or other hearing disturbances (10%), aural fullness, hyperacusis (2.5%). The hearing disturbances are accompanied by nausea and vomiting in 50–70% of cases [5,19]. The presumed pathophysiology relates to traction of the vestibulo-cochlear nerve, although an alteration of the pressure gradient between CSF and the perilymphatic fluid together with abnormal pressure in the endolymphatic/perilymphatic inner ear fluid and endolymphatic hydrops may also contribute to causing these symptoms [5].

#### 3.1.4. Ocular Findings

Most often, patients complain of photophobia (4.6%) or blurred vision and visual obscurations (4.1%) [19], symptoms which may suggest intracranial hypertension. Diplopia, described in almost 4% of patients [19], may be caused by paralysis of any of the three nerves innervating the extraocular muscles, although the abducens nerve, due to its long intracranial course, is most susceptible to traction and is most often affected (83%) [20].

#### 3.1.5. Other Cranial Nerve Symptoms

A series of symptoms ascribable to traction of various cranial nerves, such as facial numbness, facial weakness, and dysgeusia, may be reported by a small proportion of patients [25].

#### 3.1.6. Altered Mental Status

Cognitive functions are also impaired by the displacement of cranial structures progressing to brain herniation syndromes. The slowness in thinking, somnolence, lethargy (12–32%) [20], behavioral changes (disinhibition, stereotypical behaviors), together with memory disturbances may mimic fronto-temporal dementia [26] or may progress to coma in extreme cases [27].

#### 3.1.7. Motor Abnormalities

Movement disorders such as abnormal gait, ataxia, tremor, bradykinesia, dysarthria, seizures, or non-convulsive status epilepticus have been reported in different case series with variable frequencies, ranging from 1.2% [19] to 12.5% [28].

#### 3.1.8. Other Symptoms

Galactorrhea with elevated prolactin levels, reported in up to 24% of patients, is believed to be caused by brain sagging and pituitary gland hyperemia [20,29].

Cervical radiculopathy, reported in up to 6% of patients [20], usually occurs at the site of the CSF leak and is due to compression of the root by the extradural CSF collection [30].

### 3.2. Evaluation

#### 3.2.1. Lumbar Puncture

Two-thirds of patients show an opening pressure of less than 60 mm water (normal range 65–195 mm water) [8,19], but 32% have normal, and 3% of patients show even increased CSF pressure on lumbar puncture [19], highlighting the inconsistency of this finding. The CSF opening pressure does not necessarily reflect the intracranial pressure in the upright position or during postural changes and does not offer any information regarding dural compliance, which appears to play a key role in the pathophysiology of the disease, as suggested by the correlation with connective tissues disorders [31].

Examination of the CSF often reveals elevated protein content and lymphocytic pleocytosis, sometimes up to 200 cells/mmc, leading to confusion with infectious meningitis [8].

#### 3.2.2. Imaging of the Brain

Brain CT scan is often normal or may show non-specific signs, such as bilateral fluid collections (hygromas or subdural hematomas), engorged transverse venous sinuses, small ventricles, or hyperdensities in the projection of the tentorium or Sylvian fissure [5]. Therefore, it is less useful than magnetic resonance imaging (MRI) in diagnosing intracranial hypotension.

MRI is the most sensitive imaging evaluation [19], showing:

- Diffuse pachymeningeal enhancement is detected in 73–83% of cases [19,32]. The dural thickening is typically diffuse and non-nodular. It needs to be differentiated from immunoglobulin 4 (IgG4)-related pachymeningitis [33], neurosarcoidosis (pachymeningeal and/or leptomeningeal enhancement is most prominent at the skull base) [34], infectious meningitis (in tuberculous meningitis the leptomeninges is usually also involved, while in syphilitic, cryptococcal meningitis or Lyme disease the involvement of other organs and systems may provide useful clues to diagnosis [35], or a series of immune-mediated conditions, such as rheumatoid arthritis, temporal arteritis, polyangiitis with granulomatosis, in which the dural enhancement is rather nodular [32].
- Subdural fluid collections—mostly bilateral hygromas—are described in 43–50% of patients [5,19]. Hygromas occur because of the enlargement of the subdural space secondary to the loss of the CSF, while subdural hematomas may be caused by tearing of the abnormally engorged and dilated cortical veins [32]. Drainage of these collections will not resolve them successfully if the CSF leak is not identified and treated [36].
- Signs of brain sagging include flattening of the ventral pons, effacement of the prepontine and perichiasmatic cisterns, or downward displacement of the cerebellar tonsils and brain stem [5,8], which may mimic Chiari type 1 malformation. However, in SIH, the tonsils maintain normal shape and do not descend more than 5 mm below the foramen magnum [37]. Moreover, the findings associated with syringomyelia would argue for the diagnosis of Chiari type 1 malformation [32].
- Dural venous sinus engorgement appears usually as a dilation of the transverse sinuses, which is subtle and often diagnosed retrospectively, by comparison of pre- and post-treatment images [5].
- Pituitary gland hyperemia leads to enlargement of the gland and can be mistaken for a pituitary adenoma [5].
- Reduced optic nerve sheath diameter and thickness (normal—4.4 mm; reduced to a mean of 3.4 mm) is best measured on coronal T2-weighted images or with transorbital ultrasonography [38].

#### 3.2.3. Imaging of the Spine

Spinal imaging is performed in search of the CSF leak to confirm the diagnosis and help guide the treatment. Spinal MRI, myelography, or CT myelography may be chosen by different institutions, depending on the availability of the devices and the experience of physicians [32]. A consensus opinion is that a whole spinal MRI (from the cervico-cranial junction to the sacrum) with T2 fat suppression sequences should be preferred as a first-line investigation whenever possible [5]. After identifying the epidural collection via MR imaging or CT myelography, subsequent imaging with dynamic myelography, digital subtraction myelography, or dynamic CT myelography tailored to the suspected leakage site allows for precise localization of the CSF leak or the CSF-venous fistula [39].

A. MR imaging of the spine

Although MRI is available, non-invasive, and does not use ionizing radiations, it has several disadvantages, such as decreased sensitivity to low-flow leaks, inability to detect subtle bone pathology, and technical artifacts [39].

The CSF leak appears as a fluid signal within the spinal canal and outside the thecal sac, being separated in T2 weighted images by a thin dark line (the dura) from the subarachnoid space [39]. The leaked CSF may spread over several vertebral levels and exits the spinal canal through one or more neural foramina in the case of high-flow or fast leaks. Low-flow or slow leaks lead to epidural collections that usually span over a single vertebral body [39].

Other abnormalities shown by spinal MRI include [5]:

- Cervical pachymeningeal enhancement
- Non-compressive spinal epidural fluid collections
- Engorgement of the epidural venous plexus
- Meningeal diverticula, dilated nerve root sleeves, that can be nonspecific unless very large and irregular [40]
- Fluid collection in soft tissues near the C1–C2 vertebrae (but which sometimes may be a false CSF leak localizing sign)

However, precise localization of the leak is challenging in the presence of a large epidural collection [39]. Intrathecal administration of gadolinium-based contrast agents can increase sensitivity [41], but overdose of the contrast agent may lead to neurologic injury and long-term safety data are lacking [39,42,43].

B. Computed tomography imaging of the spine

CT myelography (CTM) is the most reliable test to identify the cause and site of the CSF leak [5]. It is widely available, has high spatial resolution, depicts calcified disks and osteophytes, and has excellent resolution between CSF and surrounding tissues, but uses ionizing radiation and is invasive (requiring a dural puncture) [39]. Furthermore, it is important to scan the patient as quickly as possible after the intrathecal injection of the contrast agent to capture fast leaks, a technique known as dynamic CTM [5]. The clinician should have some suspicion as to the site of the leak because the patient must be placed in a position to allow the contrast agent to cover that surface while tilting the fluoroscopy table. Radiation exposure is the main concern with the CT-based dynamic technique. In addition, the patient should be completely still during the examination, undergoing a long breath hold, which sometimes requires general anesthesia [39].

CSF-venous fistulas are missed by MR imaging and are visualized only on CTM or digital subtraction myelography (DSM). In approximately 20% of patients with initially negative spinal imaging, DSM shows a vessel filling with myelographic contrast agent originating from a nerve root sleeve [44]. On CTM, CSF-venous fistulas appear as increased attenuation of a paraspinal vein to more than 70 Hounsfield units [45,46], a finding that can easily be overlooked [47].

### 3.3. Diagnostic Criteria

According to the International Classification of Headache Disorders, 3rd edition [21], the diagnostic criteria for headaches attributed to low cerebrospinal fluid pressure are:

A. Any headache fulfilling criterion C
B. Either or both of the following:
  I. Low cerebrospinal fluid (CSF) pressure (<60 mm H_2_O)
  II. Evidence of CSF leakage on imaging

C. Headache has developed in temporal relation to the low CSF pressure or CSF leakage or led to its discovery
D. Not better accounted for by another ICHD-3 diagnosis

* Notes:

1. Headache attributed to low cerebrospinal fluid (CSF) pressure is usually but not invariably orthostatic. Headache that significantly worsens soon after sitting upright or standing and/or improves after lying horizontally is likely to be caused by low CSF pressure, but this cannot be relied upon as a diagnostic criterion.
2. Brain imaging shows brain sagging or pachymeningeal enhancement, or spine imaging (spine MRI or MRI, CT, or digital subtraction myelography) showing extradural CSF.
3. Evidence of causation may depend upon onset in temporal relation to the presumed cause, together with exclusion of other diagnoses.

### 3.4. Differential Diagnosis

#### 3.4.1. Clinical Differential Diagnosis

A series of other headache disorders have clinical similarities with SIH, but certain features may help in the differential diagnosis, as shown in Table 1.

#### 3.4.2. Imagistic Differential Diagnosis

As discussed previously, brain imaging in SIH reveals pachymeningeal enhancement, signs of brain sagging, dural venous sinus engorgement, subdural hematomas or hygromas, and pituitary enlargement. As such, a series of other disorders must be considered, as shown in Table 2.

### 3.5. Treatment

There are several treatment options for spontaneous intracranial hypotension: conservative therapy, epidural blood patch, or surgery. Because most published studies are retrospective case series reporting on one treatment modality, recommendations are based on small, uncontrolled case series and expert opinion [13].

#### 3.5.1. Conservative Therapy

Conservative therapy includes bed rest to relieve the orthostatic headache and create conditions for spontaneous closure of the CSF leak, as well as vigorous hydration [56]. The efficacy of corticosteroids is uncertain and carries the risk of side effects [5,6]. Caffeine and theophylline are sometimes recommended to increase the rate of CSF formation, but proof of efficacy is lacking [5].

The overall success rate of the conservative approach is about 28% [19]. In all, 61% of patients still experience at least moderate headache by 6 months and 50% still have symptoms after 2 years [57]. Moreover, due to the frequently delayed diagnosis, patients usually have already undergone conservative therapy by the time of presentation.

#### 3.5.2. Epidural Blood Patch

Epidural blood patch (EBP) is the most commonly performed treatment for spinal CSF leaks that fail to improve with conservative therapy. The reported response rate ranges between 36% and 90% [22,58]. It is usually performed with 10–50 mL autologous blood [5,25] and acts immediately via volume replacement. The long-lasting effect of sealing the leak is due to the interaction between the blood and pro-coagulant components in the leaking CSF, leading to the formation of a clot [5,59].

Non-targeted EBP (blind patch) can be performed in the lumbar area or at the level of the thoracolumbar junction when the site of the leak is not properly identified. Following the procedure, bed rest in the Trendelenburg position for at least 8 h is imposed, to allow blood to ascend to the CSF leak. If necessary, the procedure can be repeated twice, at least 7 days apart [5].

Targeted EBP, either with autologous blood and/or fibrin glue, may increase the response rate and is used mainly in the thoracic and upper cervical regions [58,60]. It is performed after two or three “blind” lumbar EBPs have failed [5].

Adverse events are minor and transient, consisting of back pain, radicular pain, numbness, paresthesia, tinnitus, dizziness, or bradycardia [19,61].

#### 3.5.3. Surgery

Surgery is recommended for patients who fail EBP and have well-localized CSF leaks [39]. Nerve root sleeve diverticula are treated by clipping of the root sleeve, dural repair, or epidural packing [39]. Ventral leaks are more difficult to treat surgically. A posterior transdural approach allows for the repair of the dural tear and concomitant resection of associated osteophytes [62]. Minimally invasive techniques are rapidly developing [63].

Surgery is also required for the repair of CSF-venous fistulas, since EBP is less effective in these cases [45].

#### 3.5.4. Complications of Treatment

Rebound intracranial hypertension is a complication of treatment occurring in 7–27% of patients [19]. It is due to increases in the CSF pressure above normal thresholds and manifests as headache worsening in the recumbent position, accompanied by nausea and blurring of vision. Its onset is within the first 36 h following EBP and requires measures to decrease the CSF pressure, such as head elevation, oral acetazolamide, therapeutic lumbar puncture, or even ventricular drainage [39,64].

## 4. Concluding Remarks

By presenting our spontaneous intracranial hypotension case report and the challenges encountered before reaching the correct diagnosis, together with an updated review of the clinical picture, positive and differential diagnosis, evaluation, and treatment options, we hope we have succeeded in raising awareness of this condition both among neurologists and other physicians who see patients complaining of headache. The main messages we wanted to emphasize are:

1. Despite the general belief, SIH is not a rare disease, and even the incidence rate of 5/100,000/year is believed by some physicians to be an underestimation.
2. The diagnosis is based on increased awareness and the correct interpretation of the ever-broadening spectrum of symptoms and signs, which should prompt imagistic evaluation, preferably contrast-enhanced magnetic resonance imaging of the brain.
3. Diffuse, smooth dural enhancement detected on brain MRI is highly suggestive of SIH and should be rapidly followed by spinal imaging to detect the CSF leak.
4. Although conservative treatment is the first step as therapy, it often fails to relieve the symptoms, and patients need to be referred to a neurosurgeon.
5. Failure of 2–3 non-targeted epidural blood patches indicates the need for targeted approaches (epidural blood patch or fibrin glue) or surgical repair of the dural tear.

## Figures and Tables

**Table 1:** Clinical mimics of spontaneous intracranial hypotension.

Disorders	Common Features	Distinguishing Features	Ref.
Postural orthostatic tachycardia syndrome (POTS)	Headache worsening in the upright posture	Increasing heart rate on standing from supine position with negligible changes in blood pressure	[48]
Orthostatic hypotension (etiologies: autonomic failure, hypovolemia, medication adverse effects)	Orthostatic headache, worsened by Valsalva maneuvers, + pain in the neck and shoulders (“coat-hanger” pain) caused by ischemia of the paraspinal muscles, +/− tinnitus	Fall in systolic blood pressure (>20 mm Hg) and/or diastolic blood pressure (>10 mm Hg) during standing from seating or supine position, or on the head-up tilt test	[49]
Cervicogenic headache (etiology: osteoarthritis of the atlanto-occipital junction and/or upper cervical spine)	Headache worsens in upright posture (due to axial loading of the spine) + neck pain	Usually unilateral, movement of the neck worsens neck pain; digital pressure on neck muscles may augment the neck pain	[50]
Persistent postural perceptual dizziness	Unsteadiness, nausea +/− headache in upright posture	Nausea and unsteadiness more prominent, headache occurs only occasional	[51]

**Table 2:** Radiologic mimickers of spontaneous intracranial hypotension.

Condition	Common Features	Distinguishing Features	Ref.
IgG4-related hypertrophic pachymeningitis	Thickened, enhancing dura extending into the cervical canal	Increased IgG4, fibrosis can manifest in many organs, leading to a variety of presentations	[33]
Neurosarcoidosis	Pachymeningeal enhancement, clinical picture of cranial nerve damage	Pachymeningeal and/or leptomeningeal enhancement more prominent along the skull base, increased serum angiotensin convertase	[52]
Autoimmune diseases (rheumatoid arthritis, polyangiitis with granulomatosis, temporal arteritis)	Dural thickening and enhancement	Typical serologic findings	[53]
Tuberculous meningitis	Dural thickening and enhancement	Can also affect the leptomeninges, form intracranial tuberculomas, may associate spinal tuberculous arachnoiditis	[54]
Other infectious causes of pachymeningitis (syphilis, Cryptococcus, Lyme disease)	Pachymeningeal enhancement	Affect multiple organ systems beyond the nervous system, serologic findings	[32]
Chiari malformation type 1	Cerebellar tonsillar descent	In Chiari malformation the cerebellar tonsils are inferiorly pointed and descend more than 5 mm below the foramen magnus, whereas in SIH they maintain normal shape and descent in the foramen magnus is <5 mm; midbrain descent presents in SIH	[37]
Subdural fluid collections		Mostly unilateral, occurring after trauma or in elderly, precipitated by anticoagulation, whereas in SIH they are usually bilateral, accompanied by dural enhancement and signs of brain sagging	[55]

## Data Availability

Original data are available from corresponding author upon reasonable request.
